# Reaction Time Data in Music Cognition: Comparison of Pilot Data From Lab, Crowdsourced, and Convenience Web Samples

**DOI:** 10.3389/fpsyg.2019.02883

**Published:** 2020-01-08

**Authors:** James Armitage, Tuomas Eerola

**Affiliations:** Department of Music, Durham University, Durham, United Kingdom

**Keywords:** reaction times, crowdsourcing, online experiment, prolific, PsyToolkit

## Introduction

Reaction time (RT) methods have been a mainstay of research in cognitive psychology for over a century. RT methods have been applied in domains as diverse as visual perception (e.g., Ando et al., 2002), personality traits (e.g., Robinson and Tamir, 2005), and social psychology (e.g., Wang et al., 2017). In music cognition, RT methods have been used as an indirect measure of several phenomena such as harmonic expectation (Bharucha and Stoeckig, 1986), melodic expectation (Aarden, 2003) cross modal priming (Goerlich et al., 2012), absolute pitch (Miyazaki, 1989; Bermudez and Zatorre, 2009), and emotional responses (Bishop et al., 2009).

Traditionally, reaction time data has been collected in a lab. However, recent years have seen the development of software capable of collecting accurate response time data online, for instance PsyToolkit (Stoet, 2010, 2017), PsychoPy (Peirce et al., 2019), Gorilla (Anwyl-Irvine et al., 2019), and Qualtrics' QRTEngine (Barnhoorn et al., 2015) amongst others. In the early days of web-based reaction time studies, there was considerable skepticism about the viability of RT data collected online. Despite the prevalence of software specifically designed to collect reaction time data online, and the increasing incidence of Web-based data collection, there remains a degree of caution around online reaction time studies. However, recent research (Barnhoorn et al., 2015; de Leeuw and Motz, 2016; Hilbig, 2016) suggests that online reaction time data is perhaps more trustworthy than was previously thought, but these studies have not yet involved music as stimuli.

Alongside the developments in software, recruitment of participants in online studies has been made easier by the prevalence of social media and crowdsourcing platforms such as Amazon's MTurk service and Prolific. Not surprisingly, the use of crowdsourced samples by researchers is growing rapidly (Stewart et al., 2017).

However, to the authors' knowledge (with the exception of de Leeuw and Motz, 2016) the comparisons of laboratory and online RT data have focused on descriptive measures of the RT distributions, and relatively little attention has been paid to the agreement between the RT distributions as a whole. Moreover, none of these studies considers phenomena associated with music cognition. Given the widespread use of RT methods in music cognition and the growth of crowdsourcing as a recruitment tool, the authors consider there to be a need to test the viability of online RT collection specifically in the case of music cognition.

The present data report offers the results of a response time task completed in three different contexts—in a standard lab setting (“Lab”), online recruited via “traditional” online techniques (“Web”) and crowdsourced vis Prolific.ac (“CS”). Below, we present summary data for the three data sets before testing the comparability of the three data sets on an item-by-item basis.

## Data Collection

###  Reaction Time Task and Stimuli

Data was collected using PsyToolkit (Stoet, 2010, 2017) for the lab and both online samples. PsyToolkit offers a choice of either a local installation in Linux or a browser-based version that can be used to collect data online. The PsyToolkit script used for the Lab, Web, and CS data collection was identical in all three cases. Participants completed an affective priming task in which they heard a short (~1,000 ms) extract of music (.wav files in the Lab sample; .mp3 in the Web and CS samples) before being presented with a visual target word. Participants had to classify each word as positive or negative as quickly and accurately as possible. There were eight music primes and eight target words resulting in 8 × 8 = 64 prime-target pairs. The music primes, which were drawn from Västfjäll (2001) and Eerola and Vuoskoski (2011) were controlled for valence and arousal, as were the eight target words, which were taken from Warriner et al. (2013). There were two music primes in each valence-arousal condition: 2× positive-high, 2× positive-low, 2× negative-high, and 2× negative-low. The target words followed the same valence-arousal distribution. Following the Lab data collection, it was found that one of the target words, *Lover*, was associated with significantly faster reaction times than the other words and was subsequently replaced with *Payday*. Both *Lover* and *Payday* have been excluded from the analysis below, leaving 56 prime-target pairs. Details of how the music clips were chosen and rated and more precise information regarding the procedure are included as Supplementary Material.

### Lab Study

Participants were all right-handed (Kalyanshetti and Vastrad, 2013; Hardie and Wright, 2014) with normal or corrected to normal vision and hearing; all were native English speakers and received £5 to complete the present study and a related study. Data were collected during June 2018. The experimental setup comprised a Lenovo laptop running Linux (Xubuntu 18.04) and PsyToolkit version 2.4.1. (Stoet, 2010). Including form-filling, the section of the experimental sessions relating to this task took around 10 min.

### Convenience Web Sample

The materials and procedure mirrored the lab data collection as closely as possible. However, one of the target words, *Lover*, was replaced with *Payday* as it was associated with significantly faster response times than any of the other target words. Additionally, audio files were converted to .mp3 format. Data was collected using the web-based version of PsyToolkit (version 2.5.2) (Stoet, 2017) during July 2018. The script used was identical to the script used for the Lab experiment. PsyToolkit allows researchers to restrict which type of devices are used to carry out online experiments, so we excluded tablets and mobile phones in order to maintain as much similarity with the lab setup as possible.

Participants were recruited online via Reddit, SurveyTandem (a survey exchange website where researchers complete each others studies in exchange for points; when researchers have amassed enough points, their studies are made available for other researchers to complete) and student email distribution lists at the University of Durham. Participants received no direct payment for participating, but had the option of entering a draw for a £25 Amazon voucher. As with the Lab sample, the inclusion criteria was right-handed native speakers of English with normal or corrected to normal vision and hearing.

### Crowdsourced Sample

Participants were recruited via Prolific (www.prolific.ac) and received a payment of £0.75. Owing to the similarity to a previous study that recruited via prolific, participants from this previous study were excluded from taking part. Participants were prescreened to be right-handed, native speakers of English. The stimuli and procedure were identical to those used for the convenience web sample. The PsyToolkit version was updated to 2.5.4: the differences between the versions focused on the user (i.e., researcher) interface and did not impact RT collection. Data collection took place during July 2019.

## Comparison of Data Sets

### Data Pre-treatment

Participants whose accuracy rate fell below 75% were excluded from the analysis. This resulted in no deletions from the Lab data, but six participants in the Web sample and two in the CS sample failed to reach the required accuracy threshold. For the remaining participants, timeouts and response times shorter than 250 ms were excluded from the analysis, as is common practice (e.g., Duckworth et al., 2002). To exclude upper outliers, individual participants' response time distributions were fitted with an exponentially modified Gaussian (ExGaussian) distribution (Ratcliff, 1993). Responses above the 95th percentile of each ExGaussian distribution were removed from the data set. Removal of timeouts and outliers accounted for the deletion of 5.7, 6.3, and 6.1% of responses from the Lab, Web, and CS data sets, respectively.

### Comparison of Summary Data

Following deletions, there were 32 participants (mean age = 24.0, 19 male) in the Lab sample, 33 (mean age = 25.1, 13 male) in the Web sample, and 34 (mean age = 32.7, 8 male) in the CS sample.

The three data sets are compared in accuracy, attrition rate, mean, and variance of response time in the 56 prime-target conditions. Summary data is contained in Table 1.

**Table 1:** Summary statistics for RT distributions.

**Method**	**Error rate (%)**	**Mean**	**Variance**	**Median**	**IQR**	**Timeouts (%)**
Lab	3.67	580.34	25959.44	538	161	5.75
Web	3.64	587.352	16278.84	564	133	6.28
CS	3.30	587.98	18741.63	564	140	6.14
Combined	3.53	585.30	20267.40	557	148	6.06

The mean (SD) percentage error rates for the Lab, Web, and CS samples were 3.67 (0.188), 3.64 (0.187), and 3.30 (0.179), respectively. Linear mixed effects modeling suggested that there was no significant differences in accuracy rates between the Lab, Web, and CS samples, *F*_(2, 110)_ = 0.32, *p* = 0.728. A repeated measures Anova was carried out to compare the mean response time for each target-prime pair. The test proved non-significant, *F*_(2, 54)_ = 1.883, *p* = 0.16. However, the result does perhaps suggest (non-significantly) slower mean response times in the Web and CS sample as compared to the Lab sample.

Similarly, a repeated measures Anova was carried out to compare the variances in response times for each target-prime pair. There was a highly significant difference in variances in response times between the Lab (mean Variance = 26,396) and Web (mean Variance = 16,227) or CS (mean Variance = 18,742) samples, *F*_(2, 110)_ = 26.22, *p* < 0.0001. Contrary to expectations, *post-hoc* testing indicated that variance in the Lab RTs was greater than the variance in Web or CS RTs.

### Comparison of RT Distributions

**Figure 1:**
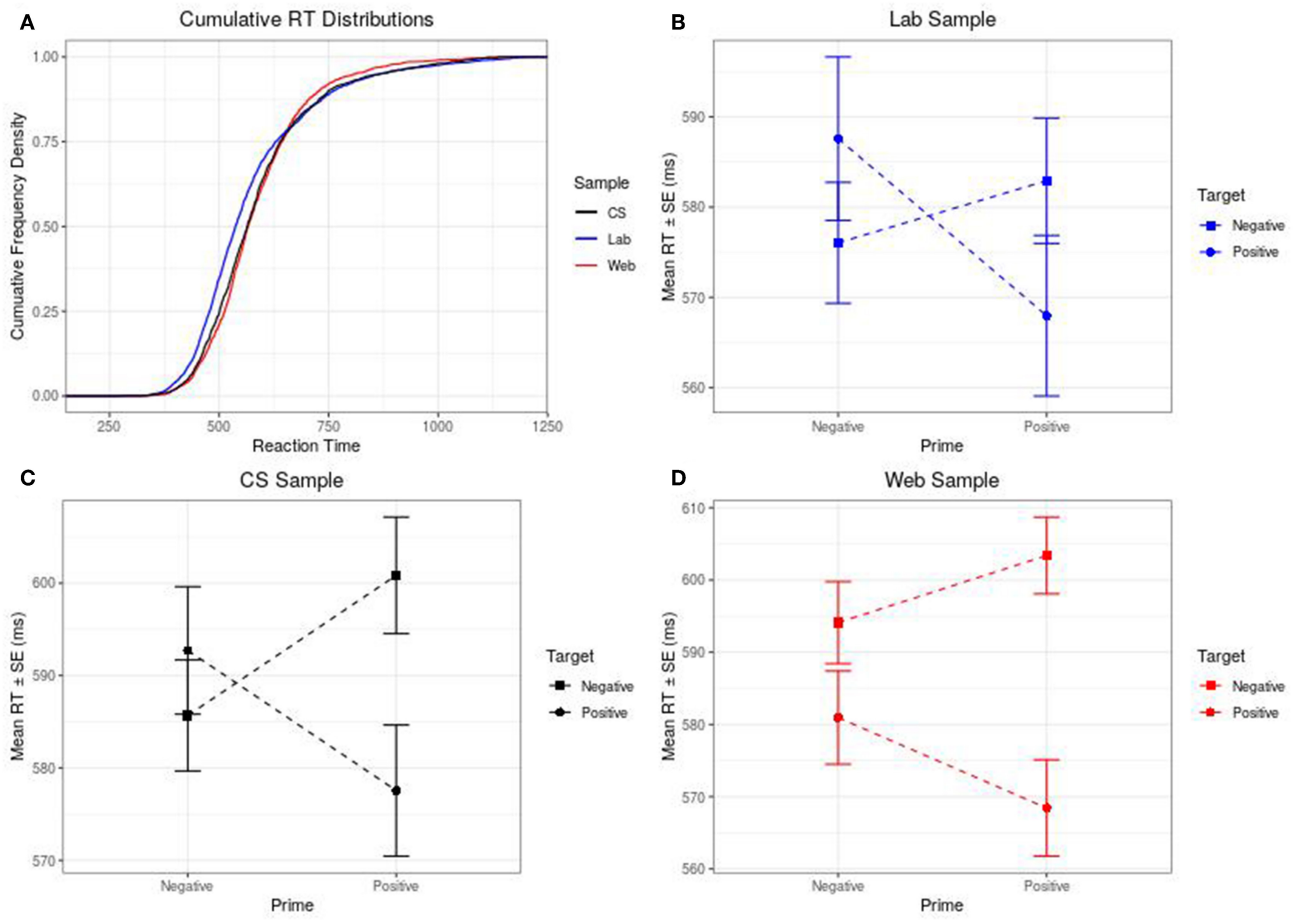
**(A–D)** Cumulative RTs & Prime Valence×Target Valence Interactions for Lab, Web, and CS samples.

In addition to the comparison of the summary data carried out above, we also carried out overall and per-item comparison of the RT distributions in the three data sets. Figure 1A shows the overall cumulative RT distributions of the three data sets.

Following the procedure set out by Voss et al. (2013) to compare response time distributions for binary choice data, incorrect responses were allocated a negative response time (for instance an incorrect answer with a response time of 450 ms was coded as –450). Next, Kolmogorov-Smirnov (KS) tests were carried out to compare the Web Convenience vs. Lab response time distributions for all 56 prime-target pairs. Eight out of the 56 results returned significant results, suggesting that, in these instances, the Web Convenience sample and the Lab sample could not be thought of as representing the same underlying RT distribution. A binomial test was carried out with *n* = 56, *r* = 8, and *p* = 0.05 to determine whether eight instances of disagreement between the Lab and Web Convenience samples is more than can be expected by chance. This test returned a significant (*p* = 0.006) result suggesting that the overall distributions of RTs for the prime-target pairs of the Web Convenience and Lab samples cannot be considered equivalent.

The same procedure was carried out to compare the RT distributions for the Lab and CS data sets. As before, we carried out KS testing to determine the goodness of fit between the two RT distributions for each prime-target pair. Two conditions yielded significant results. Binomial testing (*n* = 56, *r* = 2, *p* = 0.05) confirmed that two out of 56 conditions is below the threshold for significance (*p* = 1), suggesting a strong agreement between the Lab and CS data sets.

### Presence of Hypothesized Effects

Although reporting the results of the priming studies *per se* is outside the scope of this data report, it is important to know whether the key effect under investigation is present and consistent across all three samples (in this case the presence of congruency effects—i.e., are positive words evaluated faster when preceded by positive music than negative music, and similarly for negative words and music). To this end, the authors offer a brief account of the crucial *Prime Valence*×*Target Valence* interaction. Prior to analysis, RTs were log transformed (Whelan, 2008). Next, the transformed data were subjected to 2 (Prime Valence) ×2 (Target Valence) linear mixed effects modeling. In all three samples, the interaction was significant, although the effect is much more clearly visible in the Lab and CS samples [Lab: *F*_(1, 62)_ = 10.92, *p* = 0.002, $\eta_{p}^{2}$ = 0.15; Web: *F*_(1, 64)_ = 6.42, *p* = 0.02, $\eta_{p}^{2}$ = 0.09; CS: *F*_(1, 66)_ = 10.04, *p* = 0.002, $\eta_{p}^{2}$ = 0.13]. The difference in effect sizes is evident also in Figures 1B–D.

### Costs

A final consideration is of course cost. We have estimated the financial costs associated with the data collection based on 32 participants per sample. We carried out 32 experimental lab sessions totalling 8 h. Paying participants £2.50 per session and costing a research assistant's time at £11.40 per hour (the lowest agreed rate of pay for graduate students at Anonymous for peer review University for the academic year 2018–2019) leads to an overall cost of 32*2.50 + £11.40*8 = £171.20. For the CS sample, we paid, £0.80 per participant, and data collection took roughly 2 h. Including taxes, the additional fee to Prolific is 36%: 32*£0.75*1.36 + 2*£11.40 = £57.62. It is more difficult to estimate the cost of the Web sample. In total, the web version of the study was online for over 2 weeks, during which time it was necessary to occasionally repost the study on Reddit and check the number of responses. Furthermore, completing studies on SurveyTandem accounted for around 6 h of researcher time (costed as 6*£11.40 = £68.40). There is also, however, an important trade-off to consider: although the cost of the data was significantly lower than for the Lab sample and comparable to the CS sample, the quality of the data is somewhat poorer in terms of its agreement with the Lab data, data wastage and visibility of the hypothesized effect.

## Interpretation and Usage

The aim of this Data Report is to provide support for the concept of online collection of reaction time data. Additionally, the authors are able to point out some limitations and benefits of the three types of data collection. Researchers might also find it useful to compare the three data sets using specific measures of importance in their research.

One of the most striking differences is the difference in attrition rates between the Lab and CS samples and the convenience web sample. Data from all of the participants in the Lab sample and from 94.5% of the CS sample was viable, whereas in the Web sample data from 84.6% of participants was considered viable. It is, however, difficult to know why this may be the case. One possibility is that web participants were less motivated in the absence of a concrete financial incentive. Another is that participants in the web sample may have felt less invested in the research as they were taking part remotely and had not met the researcher in person. Another option is that error rates were higher because participants were taking part in sub-optimal conditions, so there could have been environmental distractors that influenced the error rates. A final option is that one of the sites used for recruitment operates a system whereby researchers exchange participation in surveys; researchers acquire points by participating in other researchers' studies. When they have accrued enough points, their study is in turn circulated to other researchers enrolled with the website. This comes with the risk that some researchers may have little intrinsic motivation to complete the tasks properly and allow the task to time out whilst still accruing points to allow for circulation of their own studies.

The results of the KS comparisons and visual inspection of the cumulative RT distributions suggest that, in principle, online collection of response time data can yield RT distributions that are comparable to those collected in a lab. However, much depends on the sample. In particular, the degree of alignment between the CS and Lab samples was much better than the alignment between the Web and Lab samples. It seems reasonable to assume that participants in the prolific sample were more motivated to complete the study than participants recruited via more traditional web methods, although it is not known whether this is a consequence of the fee paid to the CS sample or other factors such as curiosity or personality.

The per-target/prime pairing distributions from the Web sample differ significantly from the distributions recorded in the Lab. Given the significantly better agreement between the Lab and CS samples, it seems likely that this difference is a result of environmental or participant variables rather than browser or hardware differences.

Importantly, the hypothesized priming effect was present in all three data sets, with the caveat that the effect was much more visible in the Lab and CS samples as compared to the Web sample. Indeed, the Lab and CS samples resulted in almost identical $\eta_{p}^{2}$ effect sizes ($\eta_{p}^{2}=0.15$ and $\eta_{p}^{2}=0.13$) in the Lab and CS samples respectively, with a smaller effect size in the Web sample ($\eta_{p}^{2}=0.09$): it is noteworthy that it was still present despite the significant binomial test. One implication is that researchers who lack access to funding may still be able to collect usable data via a convenience web sample.

Overall, the data set presented here provides support for the use of web-based reaction time protocols. Researchers should, however, exercise care in their choice of participant pool. Where possible, researchers should opt for participant pools where they can be confident in the degree of motivation and engagement on the part of the participants. Moreover, researchers may wish to carry out confirmatory lab studies. The benefits of this approach extended also to faster data collection than was the case with the Web sample whilst being more cost effective than Lab data. The data presented here align with Hilbig's (2016) findings that, in principle, RT phenomena can be captured successfully online and that online RT methods can be used successfully in music cognition.

## Data Availability Statement

The raw data, stimuli, PsyToolkit code and R scripts used to analyse the RT data can be found at https://osf.io/yhsqv/?view_only=87ae6378312c4a539fd6a5316c983afb. A copy of the priming task is available at https://www.psytoolkit.org/cgi-bin/psy2.5.4/survey?s=MBxuc.

## Ethics Statement

This study was carried out in accordance with the recommendations of the Music Department Ethics Committee and approved by the same committee. In the case of the lab study, informed consent was given in writing; in the case of the online studies, informed consent was given via an online checkbox.

## Author Contributions

JA wrote the PsyToolkit code, oversaw the Lab and Web data collection, carried out the data analysis, and wrote the first draft of the manuscript. TE proposed the concept of comparing the three datasets, oversaw the CS data collection, and contributed to the authorship of the manuscript.

### Conflict of Interest

The authors declare that the research was conducted in the absence of any commercial or financial relationships that could be construed as a potential conflict of interest.

## Supplementary Material

More precise details regarding the selection of prime stimuli and procedure for the RT task are included as supplemental materials. Although strictly outside of the scope of this data report, we have reported the results of the congruency effects of interest in the priming task.
