# Supplementary material for: Reaction Time Data in Music Cognition: Comparison of Pilot Data From Lab, Crowdsourced, and Convenience Web Samples
Source: Front Psychol. 2020 Jan 8;10:2883. doi: 10.3389/fpsyg.2019.02883 (PMC6960264; doi:10.3389/fpsyg.2019.02883)
Supplement: Supplementary file 1 [file Data_Sheet_1.PDF]

Supplementary Material

1 METHOD FOR REACTION TIME (PRIMING) TASK

1.1 Materials & Stimuli

32 musical clips, drawn from the list of excerpts used for musical mood induction compiled by Västfjäll (2001) and by Eerola and Vuoskoski (2011), were previously rated by a volunteer sample (n = 42, mean age = 37). The clips were rated on a scale of 1 to 7 for valence and arousal. The 16 clips (two per condition) that most clearly represented the four conditions Positive-High, Positive-Low, Negative-High, Negative-Low were chosen as stimuli for the main experiment.

In common with Scherer and Larsen (2011), the auditory stimuli were each approximately 1000 ms in duration, where minor variation was allowed to maintain the musical integrity of the clips. Table S1 lists the musical excerpts, which are also available as an electronic appendix. The duration of 1000 ms is considered to provide the optimal trade off in being sufficiently long to induce an affective response whilst avoiding decay in the priming effect (Hermans et al., 2001; Bigand et al., 2005). Musical excerpts rather than individual chords or short progressions have been chosen to mirror the ecologically valid approach taken by Scherer and Larsen (2011).

Excerpt	Valence	Arousal
The Good Life	Positive	High
Sousa - Stars and Stripes Forever	Positive	High
Holst - The Planets: Venus, The Bringer of Peace	Positive	Low
Shine	Positive	Low
Holst - The Planets: Mars, The Bringer of War	Negative	High
Lethal Weapon 3	Negative	High
Grieg - Aase's Tod	Negative	Low
Albinoni - Adagio	Negative	Low

Table S1. Musical Excerpts

The eight target words were taken from Warriner et al. (2013) and were chosen to represent the same distribution of valence-arousal pairings as the music clips. The target words are shown in Table S2. Together, these resulted in sixty-four target-prime (8 music prime $\times$ 8 target word) pairings. Target words were presented in the centre of the screen in white 40 point arial font on a black background.

Word	Valence	Arousal
Excite	Positive	High
Lover/Payday	Positive	High
Comfy	Positive	Low
Relax	Positive	Low
Arrest	Negative	High
Fatal	Negative	High
Dismal	Negative	Low
Morgue	Negative	Low

Table S2. Target Words

1.2 Procedure

Participants completed an affective priming task, in which target words were classified as positive or negative. Each target word was preceded by an audio clip. Items consisted of a fixation cross (450 ms), followed by the music prime. 450 ms into the prime, the target word was presented in the centre of the screen. Participants had a maximum of 2000 ms to classify the target word as positive (by pressing the ”m” key) or negative (by pressing the ”z” key). Participants completed two blocks: a ten-item practice block, and a sixty-four item experimental block. During the practice block, participant received a message after each item to inform them if their response was correct (”correct!”) or incorrect (”incorrect response or too slow”).

REFERENCES

Bigand, E., Tillmann, B., Poulin-Charronnat, B., and Manderlier, D. (2005). Repetition priming: Is music special? *The Quarterly Journal of Experimental Psychology Section A* 58, 1347–1375

Eerola, T. and Vuoskoski, J. K. (2011). A comparison of the discrete and dimensional models of emotion in music. *Psychology of Music* 39, 18–49

Hermans, D., De Houwer, J., and Eelen, P. (2001). A time course analysis of the affective priming effect. *Cognition & Emotion* 15, 143–165

Scherer, L. D. and Larsen, R. J. (2011). Cross-modal evaluative priming: Emotional sounds influence the processing of emotion words. *Emotion* 11, 203

Västfjäll, D. (2001). Emotion induction through music: A review of the musical mood induction procedure. *Musicae Scientiae* 5, 173–211

Warriner, A. B., Kuperman, V., and Brysbaert, M. (2013). Norms of valence, arousal, and dominance for 13,915 English lemmas. *Behavior Research Methods* 45, 1191–1207
